# Metabolic Dysregulation and Psychosocial Stress in Patients with Schizophrenia Spectrum Disorders: A Case-Control Study

**DOI:** 10.3390/jcm9123822

**Published:** 2020-11-26

**Authors:** Błażej Misiak, Patryk Piotrowski, Jan Aleksander Beszłej, Sylwia Kalinowska, Magdalena Chęć, Jerzy Samochowiec

**Affiliations:** 1 Department of Psychiatry, Wroclaw Medical University, Pasteura 10 Street, 50-367 Wroclaw, Poland; patryk.piotrowski@umed.wroc.pl (P.P.); jabeszlej@gmail.com (J.A.B.); 2 Department of Psychiatry, Pomeranian Medical University, Broniewskiego 26, 71-460 Wroclaw, Poland; kalisy@onet.eu (S.K.); samoj@pum.edu.pl (J.S.); 3 Department of Clinical Psychology, Institute of Psychology, University of Szczecin, Krakowska 69 Street, 71-017 Szczecin, Poland; magda.chec@gmail.com

**Keywords:** psychosis, physical health, hormone, metabolic syndrome, obesity

## Abstract

Patients with schizophrenia spectrum disorders have a reduced life expectancy, which is largely the consequence of a high co-occurrence of cardiovascular diseases. To date, several intrinsic and environmental factors underlying this phenomenon have been found. However, the association with psychosocial stress has not been extensively addressed. In this study, we tested the relationship between a history of adverse childhood experiences (ACEs), lifetime stressors, perceived stress and metabolic parameters in patients with schizophrenia spectrum disorders and in healthy controls. The participants included 85 inpatients with schizophrenia spectrum disorders and 56 healthy controls. Serum levels of glucose, insulin, low- and high-density lipoproteins (LDL and HDL), triglycerides, total cholesterol and high-sensitivity C-reactive protein (hsCRP) were determined. After adjustment for potential confounding factors, patients had significantly higher levels of glucose (F = 4.856, *p* = 0.030), triglycerides (F = 4.720, *p* = 0.032) and hsCRP (F = 7.499, *p* = 0.007) as well as significantly lower levels of HDL (F = 5.300, *p* = 0.023) compared to healthy controls. There were also significant effects of interactions between diagnosis and a history of ACEs on the levels of insulin (F = 4.497, *p* = 0.036) and homeostatic model assessment of insulin resistance (HOMA-IR) (F = 3.987, *p* = 0.048). More specifically, the levels of insulin and HOMA-IR were significantly higher in the subgroup of patients with schizophrenia spectrum disorders and a positive history of ACEs compared to other subgroups of participants. No significant associations between lifetime stressors and perceived stress with metabolic parameters were found. Our findings indicate that a history of ACEs might be associated with insulin resistance in patients with schizophrenia spectrum disorders. Therapeutic strategies targeting early-life stress should be considered with early interventions that aim to manage cardiometabolic comorbidity in patients with schizophrenia spectrum disorders.

## 1. Introduction

Common risk factors for cardiovascular complications, including obesity, dyslipidemia, diabetes, hypertension and cigarette smoking, often co-occur in patients with psychotic disorders [1]. Although several mechanisms underlying this phenomenon have been identified, these abnormalities still account for reduced life expectancy in patients with schizophrenia spectrum disorders. Current interventions preventing or treating cardiometabolic comorbidities in patients with psychosis focus on modification of lifestyle and antipsychotic treatment. However, evidence regarding their efficacy is still limited [2] and there is no evidence for improvement of life expectancy over time in patients with schizophrenia [3]. 

Metabolic abnormalities already appear at the onset of psychosis, before the initiation of antipsychotic treatment. These include disrupted hormonal regulation of appetite [4], lipid profile alterations [5], indices of impaired glucose metabolism [6], hyperhomocysteinemia [7] and subclinical inflammation [8]. Possibly, some metabolic abnormalities precede the onset of psychosis [9]. Following these observations, three potential explanations were taken into consideration. Firstly, an increasing number of studies show that schizophrenia spectrum disorders and cardiometabolic risk factors might have common genetic underpinnings. For instance, on the basis of a meta-analysis, our group demonstrated that individuals with a familial high risk of psychosis may present impaired glucose tolerance [10]. Andreassen et al. [11] found that some gene variants might be related to a risk of cardiovascular complications and schizophrenia. It has been demonstrated that the polygenic risk score for schizophrenia is related to the serum levels of immune and metabolic parameters in patients with first-episode psychosis (FEP) [12]. Another explanation is that early metabolic dysregulation in psychosis reflects the effects of environmental insults acting before the onset of psychosis. Finally, interactions between genetic predisposition and environmental insults should also be considered.

Accumulating evidence shows that stressful experiences play an important role in the pathophysiology of psychosis (for review, see [13]). Several studies demonstrated that childhood and lifetime traumatic events increase susceptibility to psychotic disorders [14,15]. Stressful experiences across the lifespan can lead to overactivation of the hypothalamic–pituitary–adrenal (HPA) axis, which may impact glucose homeostasis and trigger the development of insulin resistance [16]. However, it remains largely unknown whether stress plays a role in the etiology of cardiometabolic comorbidities in patients with psychotic disorders. Two studies [17,18] found that a history of highly severe adverse childhood experiences (ACEs) is related to a higher body mass index (BMI) and greater levels of high-sensitivity C-reactive protein (hsCRP) in patients with psychosis. Moreover, it has recently been shown that a history of ACEs might account for higher levels of C-peptide and insulin in patients with FEP [19]. Another study revealed that a history of physical abuse and minority status are related to higher levels of glycated hemoglobin in FEP patients [20]. However, these two studies [19,20] did not include healthy controls, and thus it cannot be concluded whether the impact of ACEs on cardiometabolic risk is specific to patients with psychosis. In addition, the effects of recent and lifetime stressors on cardiometabolic risk factors in patients with psychosis have not been explored. In this study, we aimed to explore the association between ACEs, lifetime stressful experiences, perception of stress over the preceding month and metabolic parameters (lipid profile, glucose homeostasis and subclinical inflammation) in patients with schizophrenia spectrum disorders and in healthy controls.

## 2. Materials and Methods

### 2.1. Recruitment Procedures

Detailed recruitment procedures were described elsewhere [21,22,23]. We recruited 85 patients with schizophrenia spectrum disorders (40 patients with FEP and 45 patients with acute relapse of schizophrenia) and 56 healthy controls. The patients were enrolled at two university centers (Wroclaw and Szczecin, Poland). The following diagnoses were established according to the DSM-IV criteria: schizophrenia, schizoaffective disorder, schizophreniform disorder, brief psychotic disorder and delusional disorder. The Operational Criteria for Psychotic Illness checklist was used in diagnostic procedures [24] The majority of patients were receiving antipsychotics on the day of recruitment (*n* = 83). The treatment duration during hospitalization, when the patients were recruited, did not exceed 30 days (the mean chlorpromazine equivalent dosage was 380.6 ± 211.6 mg/day). The total dosage of antipsychotics was converted to chlorpromazine equivalents as described previously [25]. Psychopathological symptoms were assessed using the Positive and Negative Syndrome Scale (PANSS) [26], the Montgomery–Asberg Depression Rating Scale (MADRS) [27], the Young Mania Rating Scale (YMRS) [28] and the Social and Occupational Functional Assessment Scale (SOFAS) [29]. 

The healthy controls were enrolled from the local community by advertisements. They reported no first- or second-degree family members with psychotic and mood disorders. Both groups of participants were matched for age and sex. The study protocol (registration number: IUVE.A290.16.001) was accepted by the Ethics Committee at Wroclaw Medical University (Poland) and all participants signed informed consent forms for all research procedures. The study was carried out in agreement with the principles of the Declaration of Helsinki.

### 2.2. The Measures of Stress

A history of ACEs was obtained using the Childhood Experience of Care and Abuse Questionnaire (CECA.Q) [30]. It is characterized by good psychometric properties and its validity has been confirmed in patients with psychosis [31]. The CECA.Q is a semi-structured questionnaire that collects information on the history of parental loss, parental antipathy and neglect and physical abuse as well as sexual abuse at <17 years of age. In this study, a history of ACEs was operationalized as the experience of at least one of them.

Information about lifetime stressors was obtained using the List of Threatening Experiences (LTE) [32]. The LTE is a self-report that records a history of 12 stressful experiences. The number of these events was included as the measure of lifetime stress.

The Perceived Stress Scale (PSS) is a self-report that examines the level of stress over the preceding month [33]. The PSS includes 10 questions, each of which is based on a 5-point Likert scale. Answers are scored from 0 (never) to 4 (very often). A higher total PSS score indicates a higher level of perceived stress.

### 2.3. Biochemical Parameters

Morning blood samples were collected after overnight fasting. After centrifugation, serum samples were obtained. The levels of glucose, total cholesterol, high- and low-density lipoproteins (HDL and LDL), triglycerides and hsCRP were measured as described elsewhere [21]. The homeostatic model assessment of insulin resistance (HOMA-IR) was calculated according to the following formula: glucose (mg/dL) × insulin (uIU/mL)/405.

### 2.4. Data Analysis

The Mann–Whitney U test and the chi-square test were applied to compare continuous and categorical variables. Bivariate correlations were analyzed using Spearman’s rank correlation coefficients. Non-parametric bivariate tests were selected due to the non-normal distribution of data. Analysis of co-variance (ANCOVA) was used to test the effects of each group (patients vs. healthy controls) and the history of ACEs on metabolic parameters. Age, sex, BMI, cigarette smoking status, illness duration and the chlorpromazine equivalent dosage (CPZeq) were included as co-variates. Results were interpreted as significant if the *p*-value was <0.05. In case of significant interactions in the ANCOVA, post-hoc tests were performed. The Games–Howell test was used to perform post-hoc comparisons. Analyses were carried out using the Statistical Package for the Social Sciences, version 20 (SPSS Inc., Chicago, IL, USA).

## 3. Results

The patients and the healthy controls were similar in age, sex and BMI (Table 1). As expected, the level of education and the SOFAS score were significantly lower in the group of patients compared to the healthy controls. The patients had significantly higher LTE scores than the healthy controls. Unadjusted analyses revealed significantly higher levels of glucose, insulin, triglycerides and HOMA-IR in the group of patients compared to the healthy controls. In turn, the levels of HDL were significantly lower in the patients than in the healthy controls.

Results of the ANCOVA are presented in Table 2. We found significant effects of group (patients vs. healthy controls) on the levels of glucose, HDL, triglycerides and hsCRP after co-varying for the effects of potential confounding factors.

**Figure 1:**
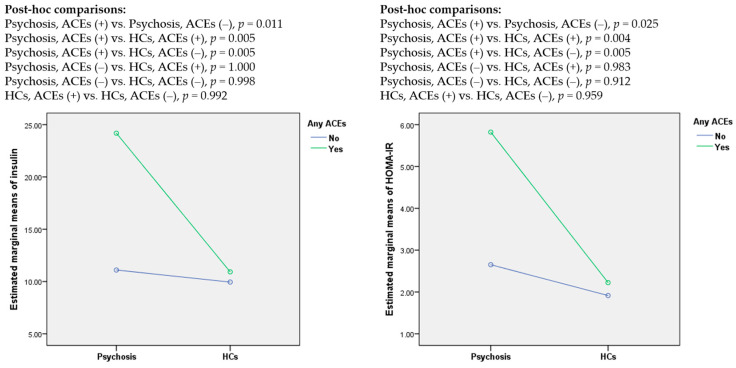
The levels of insulin and HOMA-IR with respect to a history of adverse childhood experiences (ACEs); HCs, healthy controls.

Significant effects of a history of ACEs on the levels of insulin and HOMA-IR were found. Post-hoc comparisons demonstrated significantly higher levels of insulin in patients with psychosis and a positive history of ACEs compared to other subgroups of participants (Figure 1). Other pairwise comparisons were not significant.

Correlations between perceived stress, lifetime stressors and metabolic parameters are shown in Table 3. No significant correlations between the LTE score, PSS score and metabolic parameters were found in the group of patients and healthy controls. 

## 4. Discussion

Our results confirm that patients with schizophrenia spectrum disorders show a number of metabolic abnormalities related to impaired glucose metabolism, low levels of HDL and high levels of triglycerides and hsCRP. Importantly, the patients and the healthy controls were comparable with respect to age, sex and BMI. The patients included in our sample were minimally medicated. In addition, these findings remained significant after adjustment for the effects of potential confounding factors. This is in agreement with previous studies showing high rates of metabolic syndrome and its single components in this group of patients [34,35].

Our study showed that a history of ACEs is related to higher insulin levels and greater insulin resistance in patients with schizophrenia spectrum disorders. Notably, recent and lifetime stressors were not associated with metabolic parameters tested in our study. Our recent meta-analysis revealed elevated levels of insulin in antipsychotic-naïve patients with FEP [4]. This observation suggests that metabolic dysregulation may precede the first symptoms of psychosis. Findings from this study are also concordant with the results obtained by Tosato et al. [19], who reported higher levels of insulin and C-peptide in patients with FEP. Moreover, previous studies revealed that a history of ACEs might be related to higher BMI and hsCRP levels in patients with psychosis [17,18]. One of the potential explanations underlying our results is associated with stress-induced overactivation of the HPA axis. Patients with psychotic disorders show various alterations related to the HPA axis activity, including pituitary enlargement [36], increased morning cortisol levels [37], blunted cortisol awakening response [38] and attenuated cortisol response to social stress [39]. Exposure to various psychosocial stressors may also contribute to these alterations [40]. Chronically increased levels of glucocorticoids may increase food intake, leading to the development of visceral adiposity and insulin resistance [41,42,43].

A lack of association between a history of ACEs and insulin resistance in healthy controls suggests the potential role of certain moderating factors not tested in this study. For instance, there is evidence that patients with psychotic disorders show lower levels of resilience [44] and use ineffective stress coping strategies [45,46]. Lee et al. [47] found that a higher level of childhood trauma is correlated with lower physical well-being, higher fasting insulin levels and greater insulin resistance in patients with schizophrenia. In this study, higher levels of resilience counteracted the effects of childhood trauma on mental and physical well-being. Another mechanism that may explain susceptibility to obesity and related metabolic dysregulations is associated with genetic factors. A recent genome-wide association study demonstrated that the two most significantly enriched pathways in patients with schizophrenia are associated with insulin secretion [48]. Moreover, on the basis of a Mendelian randomization study, Li et al. [49] found that higher fasting insulin levels might be a causal factor in the development of schizophrenia.

This study has some limitations that should be addressed. Firstly, our sample size was relatively small, and its representativeness might be insufficient. Secondly, the confounding effect of antipsychotics cannot be ruled out as the use of CPZeq might only partially reflect medication effects. Moreover, we did not record the total duration of antipsychotic treatment, which likely would have better reflected the cumulative load of antipsychotic treatment. Another limitation is that a history of ACEs and lifetime stressors was recorded using retrospective self-reports, and thus a recall bias should be considered. Additionally, the LTE records a limited range of psychosocial stressors across the lifespan and this study does not provide insight into potential moderating factors. Finally, the cross-sectional design limits the possibility of establishing causal associations.

In summary, our findings indicate that metabolic abnormalities in terms of higher insulin levels and greater insulin resistance can be related to a history of ACEs in adult patients with schizophrenia spectrum disorders. Lifetime and recent stressors may not be related to metabolic parameters in this group of patients. Findings from this study imply that a history of ACEs should be routinely recorded in patients with schizophrenia spectrum disorders and considered with interventions that aim to improve physical health in this population. Longitudinal studies of individuals at risk of psychosis are warranted to shed light on the direction of causality for the association reported in this study. Additional insights into causal associations can also be provided by studies that longitudinally examine mental and physical health of children and adolescents exposed to early-life stress.

## Figures and Tables

**Table 1:** General characteristics of patients and healthy controls.

	Psychosis (*n* = 85)	Healthy Controls (*n* = 56)	*p*
Age, years	37.1 ± 13.5	32.5 ± 8.2	0.120
Sex, male	52.9%	39.3%	0.124
Education, years	13.1 ± 2.8	15.8 ± 2.5	**<0.001**
BMI, kg/m^2^	25.1 ± 4.7	23.8 ± 3.5	0.074
Cigarette smoking, yes	46.2%	8.9%	**<0.001**
Any ACEs	71.2%	57.4%	0.132
LTE	5.9 ± 2.7	3.6 ± 2.2	**<0.001**
PSS	23.1 ± 6.4	22.5 ± 4.0	0.352
SOFAS	45.2 ± 16.3	96.3 ± 5.5	**<0.001**
PANSS-P	14.1 ± 5.2	-	-
PANSS-N	20.9 ± 9.3	-	-
MADRS	8.1 ± 8.1	-	-
YMRS	2.1 ± 5.0	-	-
FGAs	7.1%	-	-
SGAs	71.8%	-	-
FGAs and SGAs	20.0%	-	-
CPZeq	380.6 ± 211.6	-	-
Illness duration, weeks	314 ± 466.9	-	-
Glucose, mg/dL	90.3 ± 16.8	83.1 ± 10.6	**0.004**
Insulin, uIU/mL	20.2 ± 19.6	10.9 ± 5.5	**0.006**
HOMA-IR	4.8 ± 5.0	2.3 ± 1.3	**0.002**
LDL, mg/dL	109.3 ± 37.2	102.7 ± 34.4	0.491
HDL, mg/dL	46.9 ± 15.3	62.0 ± 16.2	**<0.001**
TC, mg/dL	182.9 ± 39.7	177.7 ± 34.1	0.698
Triglycerides, mg/dL	133.0 ± 59.2	76.8 ± 31.0	**<0.001**
hsCRP, mg/L	2.0 ± 2.6	1.6 ± 2.7	0.275

ACEs, adverse childhood experiences; BMI, body mass index; CPZeq, chlorpromazine equivalent dosage; FGAs, first-generation antipsychotics; hsCRP, high-sensitivity C-reactive protein; HDL, high density lipoproteins; HOMA-IR, homeostatic model assessment of insulin resistance; LDL, low density lipoproteins; LTE, the List of Threatening Experiences; MADRS, the Montgomery-Asberg Depression Rating Scale; PANSS-N, the Positive and Negative Syndrome Scale (subscale of negative symptoms); PANSS-P, the Positive and Negative Syndrome Scale (subscale of positive symptoms); PSS, the Perceived Stress Scale; SGAs, second-generation antipsychotics; SOFAS, the Social and Occupational Functioning Assessment Scale; TC, total cholesterol; YMRS, the Young Mania Rating Scale. Significant differences (*p* < 0.05) were marked with bold characters.

**Table 2:** Results of the analysis of co-variance testing for the effects of each group (patients vs. healthy controls) and a history of ACEs on the metabolic parameters after co-varying for potential confounding factors.

	Glucose	Insulin	HOMA-IR	LDL	HDL	TC	Triglycerides	hsCRP
Group	**F = 4.856** ***p* = 0.030**	F = 1.585 *p* = 0.245	F = 3.847 *p* = 0.053	F = 0.025 *p* = 0.875	**F = 5.300** ***p* = 0.023**	F = 0.008 *p* = 0.928	**F = 4.720** ***p* = 0.032**	**F = 7.499** ***p* = 0.007**
ACEs	F = 0.352 *p* = 0.554	**F = 5.960** ***p* = 0.016**	**F = 5.586** ***p* = 0.020**	F = 0.753 *p* = 0.388	F < 0.001 *p* = 0.985	F = 0.896 *p* = 0.346	F = 1.688 *p* = 0.197	F = 0.004 *p* = 0.947
Group × ACEs	F = 0.266 *p* = 0.607	**F = 4.497** ***p* = 0.036**	**F = 3.987** ***p* = 0.048**	F = 0.214 *p* = 0.645	F = 0.001 *p* = 0.972	F = 0.886 *p* = 0.349	F = 1.547 *p* = 0.217	F = 0.210 *p* = 0.647
Age	**F = 4.623** ***p* = 0.034**	F = 0.454 *p* = 0.502	F = 0.087 *p* = 0.769	**F = 21.688** ***p* < 0.001**	F = 0.151 *p* = 0.699	**F = 22.183** ***p* < 0.001**	F = 2.768 *p* = 0.099	F = 0.045 *p* = 0.832
Sex	F = 0.001 *p* = 0.977	F = 1.917 *p* = 0.169	F = 1.289 *p* = 0.259	F = 0.341 *p* = 0.561	**F = 4.094** ***p* = 0.046**	F = 0.051 *p* = 0.822	F = 2.955 *p* = 0.089	F = 0.361 *p* = 0.549
BMI	F = 1.013 *p* = 0.317	F = 0.589 *p* = 0.445	F = 0.814 *p* = 0.369	F = 1.761 *p* = 0.188	**F = 12.002** ***p* = 0.001**	F = 0.081 *p* = 0.777	**F = 6.547** ***p* = 0.012**	F = 0.848 *p* = 0.359
CPZeq	F = 0.954 *p* = 0.331	F = 0.052 *p* = 0.821	F = 0.004 *p* = 0.951	F = 2.371 *p* = 0.127	F = 0.009 *p* = 0.923	F = 3.254 *p* = 0.074	F = 2.606 *p* = 0.110	**F = 4.047** ***p* = 0.047**
Illness duration	F = 0.073 *p* = 0.787	F = 0.010 *p* = 0.920	F = 0.006 *p* = 0.937	**F = 9.096** ***p* = 0.003**	F = 0.020 *p* = 0.888	**F = 7.628** ***p* = 0.007**	F = 0.005 *p* = 0.945	**F = 4.425** ***p* = 0.038**
Cigarette smoking	F = 0.001 *p* = 0.973	F = 0.075 *p* = 0.785	F = 0.168 *p* = 0.683	F = 0.475 *p* = 0.492	F = 2.240 *p* = 0.138	F = 1.620 *p* = 0.206	F = 0.012 *p* = 0.912	F = 1.535 *p* = 0.218

ACEs, adverse childhood experiences; BMI, body mass index; CPZeq, chlorpromazine equivalent dosage; hsCRP, high-sensitivity C-reactive protein; HDL, high density lipoproteins; HOMA-IR, homeostatic model assessment of insulin resistance; LDL, low density lipoproteins; TC, total cholesterol. Significant effects (*p* < 0.05) were marked with bold characters.

**Table 3:** Correlations between perceived stress, lifetime stressors and metabolic parameters.

	LTE		PSS	
	Psychosis	HCs	Psychosis	HCs
Glucose	r = −0.087	r = 0.028	r = 0.138	r = −0.129
Insulin	r = −0.013	r = −0.045	r = 0.059	r = 0.065
HOMA-IR	r = −0.036	r = −0.049	r = 0.105	r = 0.022
LDL	r = 0.061	r = 0.189	r = 0.027	r = 0.133
HDL	r = −0.002	r = −0.050	r = −0.038	r = 0.152
TC	r = 0.113	r = 0.260	r = −0.026	r = 0.181
Triglycerides	r = 0.182	r = −0.021	r = −0.014	r = −0.067
hsCRP	r = 0.151	r = 0.061	r = 0.083	r = −0.052

*p* > 0.05, ACEs, adverse childhood experiences; BMI, body mass index; CPZeq, chlorpromazine equivalent dosage; hsCRP, high-sensitivity C-reactive protein; HDL, high density lipoproteins; HOMA-IR, homeostatic model assessment of insulin resistance; LDL, low density lipoproteins; LTE, the List of Threatening Experiences; PSS, the Perceived Stress Scale; TC, total cholesterol.
